# Development of an evaluation framework for a lifestyle management model among Chinese MASLD patients under the concept of physical and medical integration

**DOI:** 10.3389/fpubh.2026.1877424

**Published:** 2026-06-24

**Authors:** Wenjing Yan, Lina Wang, Xiaoyu Yang, Yanjun Zhang

**Affiliations:** 1 School of Physical Education, Shanxi Normal University, Taiyuan, Shanxi, China; 2 Department of Competitive Sports, Beijing College of Sports, Beijing, China; 3 Pharmacy Department, Handan First Hospital, Handan, Hebei, China; 4 Shanxi Bethune Hospital, Shanxi Academy of Medical Sciences, Third Hospital of Shanxi Medical University, Tongji Shanxi Hospital, Taiyuan, Shanxi, China

**Keywords:** health promotion, lifestyle intervention, management model, MASLD, physical and medical integration

## Abstract

**Objective:**

Metabolic dysfunction-associated steatotic liver disease (MASLD), formerly known as non-alcoholic fatty liver disease (NAFLD), has emerged as a significant public health threat in China. Under the national “physical and medical integration” framework—which advocates collaborative efforts between healthcare and sports science—there is an urgent need to develop a scientific and practical evaluation system to standardize lifestyle management programs for MASLD patients and support clinical implementation of evidence-based lifestyle interventions.

**Methods:**

Building on prior research identifying factors influencing adherence to healthy lifestyles among Chinese MASLD patients, we employed a mixed-methods approach combining in-depth interviews and a two-round Delphi survey with experts from exercise science, public health, health management, exercise prescription for chronic diseases, and hepatology. The Analytic Hierarchy Process (AHP) was used to determine indicator weights and finalize the evaluation framework.

**Results:**

Six experts participated in in-depth interviews, and 12 experts completed two rounds of Delphi questionnaires (response rates: 100% and 91.7%; authority coefficients: 0.90 and 0.89). The final evaluation system comprises three dimensions: (1) Process evaluation—6 first-level indicators (e.g., organizational structure, personnel configuration, supporting facilities) with 12 second-level indicators; (2) Effect evaluation—3 first-level indicators (patient participation, lifestyle improvement, follow-up status) with 5 second-level indicators; and (3) Outcome evaluation—2 first-level indicators (disease control, health-related physical fitness improvement) with 4 second-level indicators. Kendall's W coefficients indicated statistically significant consensus (first-level: 0.23 and 0.20; second-level: 0.22 and 0.26; all *P* < 0.05). AHP-derived weights were 0.50 for process, 0.25 for effect, and 0.25 for outcome. All consistency ratios (CR) were below 0.1, confirming the validity of the weighting.

**Conclusion:**

This study successfully developed a multidimensional, reliable, and practice-oriented evaluation system for lifestyle management of MASLD patients under the integration of medicine and physical activity framework. The system provides a standardized tool to assess and improve the quality of integrated lifestyle interventions in clinical settings, offering actionable guidance for delivering coordinated, patient-centered care for MASLD.

## Introduction

Metabolic dysfunction-associated steatotic liver disease (MASLD), formerly known as non-alcoholic fatty liver disease (NAFLD), has become the most common chronic liver disease (1). With the global rise in obesity and type 2 diabetes mellitus, MASLD has become the most prevalent chronic liver disease worldwide. It not only significantly increases the risk of end-stage liver disease but is also strongly associated with extrahepatic complications, particularly cardiovascular diseases (2, 3). Epidemiological evidence indicates that the annual incidence of MASLD in Asia reaches 52.34 per 1,000 person-years—substantially higher than in Western populations (4). Although historically considered a condition of older adults, emerging data reveal a rapidly growing burden among individuals aged 15 to 49 years (4). Critically, modifiable risk factors such as obesity, sedentary behavior, poor dietary habits, and metabolic syndrome are highly prevalent and increasingly common in this age group (5). Given the specific indications and cost of these medications, lifestyle management remains the core strategy for controlling the disease at the public health level (6). In China, rapid economic development and the westernization of lifestyles have driven a sharp increase in MASLD prevalence, establishing it as a major public health challenge. Beyond clinical complexity, MASLD imposes substantial economic costs through healthcare expenditures and productivity loss, while markedly reducing patients' health-related quality of life (HRQoL) (7). Consequently, translating evidence-based lifestyle recommendations into structured, sustainable, and scalable clinical services remains a critical bottleneck in MASLD prevention and control.

In recent years, the concept of physical and medical integration—a national strategy in China promoting deep collaboration between sports science and healthcare systems—has gained increasing policy and practical attention (8, 9). This approach aligns well with the behavior-centered nature of MASLD management and offers a promising framework for developing systematic, replicable lifestyle intervention models (10–12). However, in most current clinical settings, lifestyle advice for MASLD patients remains limited to brief verbal recommendations from physicians. There is a notable absence of integrated service models involving coordinated input from both medical and exercise professionals, standardized protocols, or technical support. Moreover, validated evaluation tools and policy guidance for such integrated services are lacking (13). While some studies have explored assessment frameworks in the broader contexts of health management or physical-medical integration (14, 15), none have developed a disease-specific, operational evaluation system tailored to metabolic liver diseases like MASLD.

Building on this gap and informed by our prior research on determinants of lifestyle adherence among Chinese MASLD patients, this study aims to develop a comprehensive evaluation framework for a lifestyle management model under the concept of physical and medical integration. Using in-depth expert interviews, a two-round Delphi survey, and the Analytic Hierarchy Process (AHP), we constructed a multidimensional system spanning process, effect, and outcome domains. By integrating core elements from both medical and exercise science disciplines and emphasizing real-world applicability, this framework is designed to “drive improvement through evaluation,” facilitating the transition from fragmented, *ad hoc* advice to standardized, collaborative, and assessable lifestyle services. Ultimately, it seeks to provide methodological support and a practical tool for enhancing MASLD prevention and high-quality chronic disease management at the primary care level.

## Methods

## Expert interviews and Delphi survey

Based on a systematic review of domestic and international literature and an initial conceptual framework for a lifestyle management model for MASLD patients under the concept of physical and medical integration, the research team developed a semi-structured interview guide to conduct in-depth interviews with domain experts. These interviews aimed to refine the model's components and inform the development of evaluation indicators. Subsequently, a two-round Delphi survey was conducted to optimize and validate the preliminary indicator system. The Delphi method is a well-established consensus-building technique widely used in medicine, public health, and health services research for developing evaluation frameworks. Prior evidence suggests that involving 10–15 experts ensures sufficient representativeness and reliability of results (16). Experts were selected according to the following criteria: (1) holding a mid-level or higher professional technical title; (2) having at least 5 years of work experience in hepatology, exercise prescription, physical and medical integration, or health management; and (3) expressing interest in and willingness to participate in this study on lifestyle management for MASLD patients. A total of 12 experts from disciplines including exercise science, public health, health management, exercise prescription for chronic diseases, and hepatology were invited to participate in the Delphi survey. Six of these experts had also taken part in the initial in-depth interviews. Demographic and professional characteristics of the expert panel are presented in Table 1.

**Table 1:** Basic information of surveyed experts.

No.	Code	Age	Highest degree	Professional title	Years of experience	Research focus	Affiliation
1	A	57	PhD	Chief Physician	>30	Cardiovascular diseases	Haidian Hospital, Beijing
2	B	53	PhD	Chief Physician	>30	MASLD diagnosis and treatment	Beijing You'an Hospital, Capital Medical University
3	C	52	PhD	Chief Physician	20–30	Orthopedic surgery	Beijing Hospital
4	D	47	Bachelor's	Chief Nurse	20–30	Clinical nursing; Chronic disease health management	Beijing You'an Hospital, Capital Medical University
5	E	48	Master's	Associate Chief Physician	11–20	Health management	The First Affiliated Hospital of PLA General Hospital (301 Hospital)
6	F	44	Master's	Intermediate-level Professional	11–20	Clinical nutrition	Beijing You'an Hospital, Capital Medical University
7	G	53	PhD	Professor	>30	Community-based physical and medical integration; Metabolomics	Xiamen University of Technology
8	H	50	PhD	Professor	20–30	Exercise and physical fitness	Beijing Sport University
9	I	52	PhD	Professor	20–30	Sports medicine; Exercise rehabilitation for chronic diseases	Beijing Sport University
10	J	48	PhD	Professor	>30	Constitution science (Physical constitution)	Beijing Normal University
11	K	35	PhD	Lecturer	5–10	Exercise-based health management	Beijing Sport University
12	L	34	PhD	Lecturer	5–10	Exercise-based health management	Beijing Sport University

## Expert interviews: content and implementation

Semi-structured expert interviews were conducted to address two core objectives: (1) Theoretical appropriateness—specifically, whether grounding the lifestyle management model for patients with MASLD in the concept of physical and medical integration within the PRECEDE–PROCEED model (a well-established framework in health promotion and behavioral intervention design) was conceptually justified; and (2) Refinement of model content, with particular attention to key operational components, including management strategies, implementation procedures, organizational structure, staffing arrangements, supporting infrastructure, and evaluation mechanisms. The interview guide is provided in Table 2. Prior to each interview, researchers contacted participating experts to explain the study aims and procedures. Written informed consent was obtained, and background materials—including a schematic of the preliminary model—were shared in advance to facilitate a shared understanding of the discussion objectives. During the interviews, researchers maintained a neutral stance and avoided leading or suggestive questioning. With participants' permission, all interviews were audio-recorded. Transcripts were generated immediately following each session and subjected to thematic analysis to identify actionable insights that informed subsequent iterations of the model.

**Table 2:** Expert interview questions.

No.	Question
1	Is the theoretical foundation appropriate? This model is grounded in the PRECEDE–PROCEED model (also known as the Green Model), developed by Lawrence W. Green, a leading figure in health education. The model integrates multiple behavioral change theories and provides a systematic, sequential framework for designing, implementing, and evaluating health education and promotion programs. Over four decades, it has served as a cornerstone of public health practice, guiding efforts in health education and promotion through five diagnostic phases (social diagnosis, epidemiological diagnosis, behavioral and environmental diagnosis, educational and organizational diagnosis, management and policy diagnosis), one implementation phase, and three evaluation phases (process evaluation, impact evaluation, outcome evaluation). Do you consider this theoretical foundation suitable for developing a lifestyle management model under the concept of physical and medical integration?
2	Is the overall framework of the model appropriate? Specifically, is it reasonable to categorize the lifestyle management approach into three core components: exercise intervention, dietary intervention, and follow-up management?
3	Are the organizational structure, staffing arrangements, and hardware facilities adequately designed?
4	Exercise intervention implementation:
	(1) Is the pre-exercise health screening appropriate? (e.g., screening for contraindications and cardiovascular risks)
	(2) Is the physical fitness assessment plan appropriate? (e.g., selection and scheduling of assessment items)
	(3) Is the design of the exercise intervention program appropriate? (e.g., phased delivery of different exercise content)
	(4) Is the content of exercise guidance appropriate? (e.g., delivery of knowledge and skills; are there any additional topics needed for health education?)
5	Dietary intervention implementation: Is the content of dietary guidance appropriate? (e.g., delivery of nutritional knowledge and skills)
6	Follow-up management implementation: Do you consider the follow-up process design reasonable?
7	Are there any other components or aspects that should be added?
8	Quality evaluation:
	(1) The theoretical framework used in this study is the classic PRECEDE–PROCEED model, which consists of three main stages. The evaluation stage includes process evaluation, impact evaluation, and outcome evaluation. Process evaluation refers to assessing all aspects of program implementation from start to finish—such as project design, implementation procedures, staff performance, etc. Impact evaluation assesses changes in target populations' health status and behaviors after intervention implementation. Outcome evaluation focuses on changes in quality of life and long-term health outcomes. Based on this framework, we have constructed an evaluation indicator system. Do you consider this theoretical application appropriate for building a quality assessment system for lifestyle interventions under the concept of physical and medical integration?
	(2) Do you consider it reasonable to use exercise, nutrition, follow-up, and management to reflect process evaluation? Should any adjustments or additions be made? Do you consider using cognition and behavior to reflect impact evaluation? Any further suggestions?
	(3) Do you consider it reasonable to evaluate outcomes through health outcomes, physical fitness, cognitive-behavioral changes, and satisfaction? Should any adjustments or additions be made?

## Delphi survey instrument development

Following the literature review and expert interviews, the research team synthesized the findings to draft an initial set of evaluation indicators, which formed the basis of the first-round Delphi questionnaire. The instrument comprised three sections: (1) a brief introduction outlining the study rationale, theoretical foundation, and preliminary work; (2) a list of candidate indicators, rated by experts using a 5-point Likert scale (5 = extremely important, 4 = important, 3 = moderately important, 2 = slightly important, 1 = not important), along with an open-ended section for suggestions on modifications or new items; and (3) a demographic and judgmental basis form collecting information on gender, age, professional title, years of experience, research focus, highest educational degree, self-rated familiarity with the topic, and sources of judgment. The second-round questionnaire was revised based on feedback from Round 1. It included: (1) an updated indicator list, clearly displaying each item's mean score, modification status, origin of newly added items, and representative expert comments to facilitate informed re-evaluation; and (2) the same demographic and judgmental basis form as in Round 1. Both rounds were distributed via email or WeChat, with a 2-week response window. A single reminder was sent after 1 week to non-respondents to improve response rates. After each round, the research team revised the indicator set based on predefined criteria, expert comments, and internal discussion, iterating until consensus stabilized and no substantive new suggestions emerged.

## Data analysis

Data were entered and analyzed using Microsoft Excel 2016 and SPSS version 26.0. Descriptive statistics—including means, standard deviations, medians, and coefficients of variation (CV)—were calculated for all indicator ratings. Expert panel performance was assessed through response rate (as a measure of expert engagement), authority coefficient (Cr), and Kendall's coefficient of concordance (W) to evaluate consensus. In the absence of universally accepted Delphi inclusion thresholds, we adopted criteria from prior studies (17, 18): (1) at least 70% of experts rated an indicator as “moderately important” or higher (score ≥ 3); and (2) the median importance score fell between 4 and 5. Upon finalizing the indicator system, the Analytic Hierarchy Process (AHP) was applied using yaahp software to derive hierarchical weights. Judgment matrices were constructed based on aggregated expert ratings, and consistency ratios (CR) were calculated to validate weighting reliability (CR < 0.1 indicating acceptable consistency). This process yielded a logically coherent, structurally sound, and practice-oriented evaluation framework for lifestyle management of MASLD patients under the physical and medical integration paradigm.

## Result

### Expert response rate and authority coefficient

The expert response rate was used as an indicator of panel engagement (response rate = number of experts who completed the questionnaire / total number of invited experts × 100%). In Round 1, all 12 invited experts returned completed questionnaires (response rate: 100%); in Round 2, 11 out of 12 experts responded (response rate: 91.7%). The authority coefficient (Cr), calculated as the average of experts' self-rated familiarity with the topic and the quality of their judgmental basis, was 0.90 in Round 1 and 0.89 in Round 2. According to established methodological guidance, a Cr value above 0.7 indicates high expert authority; thus, the panel in this study demonstrated strong credibility, supporting the reliability of the findings.

### Degree of consensus among experts

Consensus among experts was assessed using the coefficient of variation (CV) and Kendall's coefficient of concordance (W). In Round 1, Kendall' s W was 0.23 for first-level indicators and 0.22 for second-level indicators (both *P* < 0.05). In Round 2, W values were 0.20 and 0.26, respectively (both *P* < 0.05). These statistically significant results indicate a satisfactory level of agreement among experts regarding the relative importance of the proposed indicators.

## Evaluation framework for the lifestyle management model under physical and medical integration

Building on a systematic review of the literature and insights from preliminary expert interviews, the research team developed an initial evaluation framework for a lifestyle management model for MASLD patients under the concept of physical and medical integration. The framework is structured around three core dimensions—process, effect, and outcome—and encompasses key components such as organizational structure, staffing, supporting facilities, assessment protocols, intervention delivery, and outcome monitoring. Following two rounds of Delphi consultation, the indicator system was refined and finalized into a coherent and comprehensive evaluation tool. Detailed results from the second-round Delphi survey are presented in Table 3.

**Table 3:** Results of the expert survey on the evaluation indicator system.

Dimension	First-level indicator	Second-level indicator	Mean score	Standard deviation	Coefficient of variation (CV)	Median	Suggestion
Process evaluation	Organizational structure	Department composition	4.36	0.67	0.15	4	Adjust
		Institutional development	4.55	0.52	0.11	5	——
	Staffing arrangement	Personnel composition	4.45	0.52	0.12	4	Adjust
		Staff training	4.45	0.69	0.15	5	——
	Supporting facilities	Data management system configuration	4	0.63	0.16	4	——
		Physical fitness testing facility configuration	4.64	0.51	0.11	5	——
		Safety protection facility configuration	4.55	0.52	0.11	5	——
	Testing and assessment implementation	Health screening implementation	4.82	0.41	0.08	5	Adjust
		Physical fitness testing implementation	4.73	0.47	0.1	5	Adjust
	Intervention plan formulation	Exercise prescription formulation	4.64	0.51	0.11	5	Adjust
cline3-8		Dietary intervention plan formulation	4.64	0.51	0.11	5	Adjust
	Implementation and supervision	Patient satisfaction rate	4.18	0.75	0.18	4	——
Effect evaluation	Patient engagement in lifestyle management	Exercise participation rate	4.45	0.69	0.15	5	——
		Dietary participation rate	4.36	0.67	0.15	4	——
	Improvement in patient lifestyle	Exercise adherence	4.55	0.69	0.15	5	——
		Dietary adherence	4.45	0.69	0.15	5	——
	Patient follow-up status	Patient follow-up rate	4.27	0.65	0.15	4	——
Outcome evaluation	Improvement in health-related physical fitness	Body composition improvement rate	4.36	0.51	0.12	4	Adjust
		Muscle strength improvement rate	3.82	0.75	0.2	4	——
		Cardiovascular endurance improvement rate	4.09	0.7	0.17	4	——
	Disease control outcomes	MASLD improvement rate	4.64	0.51	0.11	5	Adjust
		Blood glucose improvement rate	3.91	0.7	0.18	4	Delete
		Blood lipid improvement rate	4.36	0.67	0.15	4	Delete

Within the evaluation framework for lifestyle management of MASLD patients under the concept of physical and medical integration, the weights assigned to the three first-level dimensions—process evaluation, effect evaluation, and outcome evaluation—were 0.50, 0.25, and 0.25, respectively. All pairwise comparison matrices yielded consistency ratios (CR) below the threshold of 0.1 based on the maximum eigenvalue method, indicating acceptable consistency and validating the reliability of the derived weights. The complete indicator structure and corresponding weights at all levels are presented in Table 4.

**Table 4:** Final evaluation indicator system and corresponding weights.

Dimension	Weight	First-level indicator	Relative weight	Absolute weight	Second-level indicator	Relative weight	Absolute weight
Process evaluation	0.5	Organizational structure	0.07	0.04	Institutional development	0.67	0.01
					Department composition	0.33	0.02
		Staffing arrangement	0.11	0.06	Personnel composition	0.5	0.03
					Staff training	0.5	0.03
		Supporting facilities	0.11	0.06	Physical fitness testing facility configuration	0.55	0.01
					Safety protection facility configuration	0.34	0.03
					Data management system configuration	0.11	0.02
		Testing and assessment implementation	0.32	0.16	Health screening implementation	0.67	0.11
					Physical fitness testing implementation	0.33	0.05
		Intervention plan formulation	0.17	0.09	Exercise prescription formulation	0.5	0.04
					Dietary intervention plan formulation	0.5	0.04
		Implementation and supervision	0.22	0.11	Patient satisfaction rate	1	0.11
Effect evaluation	0.25	Patient engagement in lifestyle management	0.6	0.15	Exercise participation rate	0.5	0.08
					Dietary participation rate	0.5	0.08
		Improvement in patient lifestyle	0.2	0.05	Exercise adherence	0.67	0.03
		Dietary adherence	0.33	0.02
		Patient follow-up status	0.2	0.05	Patient follow-up rate	1	0.05
Outcome evaluation	0.25	Disease control outcomes	0.5	0.13	MASLD improvement rate	1	0.13
		Improvement in health-related physical fitness	0.5	0.13	Body composition improvement rate	0.61	0.08
					Cardiovascular endurance improvement rate	0.27	0.02
					Muscle strength improvement rate	0.12	0.03

## Discussion

This study, grounded in the concept of physical and medical integration, developed the first systematic evaluation framework specifically designed for lifestyle management among patients with MASLD. Structured around the logic of process–effect–outcome, the framework comprises 11 first-level and 21 second-level indicators. Weights were rigorously determined through a two-round Delphi consultation and the Analytic Hierarchy Process (AHP), with all pairwise comparison matrices yielding CR below 0.1, confirming both structural validity and weighting reliability. In contrast to existing studies that predominantly focus on macro-level service models or generic health management frameworks, our framework offers three distinctive strengths: disease specificity—tailored to the metabolic pathophysiology and intervention needs of MASLD; interprofessional collaboration—integrating expertise from both medical and exercise science domains; and clinical operability—with concrete, actionable indicators and clear implementation pathways. Given the absence of approved pharmacotherapies for MASLD and the universal recommendation of lifestyle modification as first-line treatment in international guidelines, this evaluation tool provides a practical, assessable, and scalable mechanism to monitor and improve the quality of lifestyle-based care for a chronic condition fundamentally dependent on sustained behavioral change.

To date, evaluation research on lifestyle management for chronic diseases within the context of physical and medical integration remains limited. Existing studies have primarily focused on broad service frameworks or macro-level standardization. For instance, Chunyan et al. (19) proposed a three-subsystem structure—covering foundational standards, service support, and service delivery—from a service standardization perspective. Jia et al. (20) emphasized that integrated health service systems should achieve multi-dimensional alignment across institutional, organizational, professional, and service domains, with an increasing emphasis on a “person-centered” care philosophy. Xudong (21) developed a health management evaluation framework encompassing health behaviors, literacy, and satisfaction, yet did not foreground physical activity as a core intervention component. Xueli and Xiuhan (14) applied the Delphi method to construct evaluation indicators for pilot demonstration zones of physical and medical integration, while Xiaohong and Jianjun (15) proposed a comprehensive service framework comprising five systems: exercise safety, outcome evaluation, guidance, organization, and support—particularly highlighting the safety, effectiveness, and sustainability of exercise interventions. Nevertheless, most of these efforts remain at the theoretical or regional pilot stage and lack disease-specific, fine-grained evaluation tools tailored to particular chronic conditions—especially metabolic liver diseases such as MASLD. Building upon this foundation, our study represents a significant advancement: by focusing on MASLD—a highly prevalent and increasingly severe metabolic liver disorder—we deeply integrate exercise science (e.g., exercise prescription, physical fitness assessment) with hepatology care and nutritional management to develop the first evaluation framework that is simultaneously disease-specific, interprofessionally collaborative, and clinically operational. This framework not only directly addresses the core therapeutic need for structured lifestyle intervention in MASLD but also bridges a critical gap in translating the concept of physical and medical integration into practical, evidence-based chronic disease management. It thus provides both methodological grounding and an actionable pathway for implementing integrated care models in real-world settings.

The evaluation framework developed in this study is firmly grounded in both clinical evidence and public health imperatives. Currently, no pharmacological therapy has been approved specifically for MASLD, and international guidelines uniformly recommend lifestyle interventions—centered on dietary modification, regular physical activity, and weight management—as first-line treatment (22–24). However, the real-world situation remains concerning: most MASLD patients exhibit very low levels of physical activity (25), and healthcare systems widely lack standardized, integrated care pathways (26). Lazarus et al. (27), through a global Delphi consensus, explicitly highlighted that MASLD—representing the hepatic manifestation of metabolic syndrome—exerts multisystem effects yet has long been neglected in public health policy, underscoring the urgent need for interdisciplinary, integrated care models. Ruissen et al. (28) further emphasized that MASLD is fundamentally a “cardiometabolic-hepatic” multimorbidity, particularly in patients with advanced fibrosis, requiring coordinated management by multidisciplinary teams (MDTs) comprising hepatologists, endocrinologists, cardiologists, dietitians, and exercise specialists. Our proposed evaluation framework directly operationalizes this emerging consensus. The process dimension focuses on interprofessional collaboration through organizational structure, staff qualifications, facility readiness, and standardized intervention protocols; the effect dimension captures patient engagement, behavioral adherence, and continuity of follow-up; and the outcome dimension directly measures improvements in disease status and health-related physical fitness. Collectively, this three-tiered logic aligns closely with the core principles of modern chronic disease management: patient-centered care, behavior change as the primary goal, and multidisciplinary collaboration as the enabling mechanism. By doing so, the framework provides a systematic tool to translate evidence-based lifestyle recommendations into implementable, monitorable, and evaluable clinical services within the context of physical and medical integration.

Furthermore, the design of our evaluation framework draws extensively on successful models of integrated chronic care. A systematic review by Fuchs et al. (29) of Germany's Disease Management Programs (DMPs) for type 2 diabetes demonstrated that structured interventions significantly improve process quality—such as adherence to regular monitoring and clinical guidelines—and show early evidence of long-term mortality reduction. Similarly, the World Health Organization (WHO) recommends “embedding” MASLD care within existing diabetes or obesity management pathways in resource-constrained settings, leveraging shared screening tools, risk stratification models, and intervention platforms to achieve efficient integration and cost-effectiveness (30). Our process-level indicators—such as “whether health screening includes assessment of exercise contraindications and cardiovascular risk” and “whether physical activity or dietary plans specify intensity levels and safety precautions”—operationalize this “embedded integration” strategy, ensuring that lifestyle interventions are both safe and individualized. Moreover, Dyson et al. (31) advocate a risk-stratified approach to MASLD management: early-stage disease should be managed primarily through lifestyle modification, while advanced cases require pharmacotherapy and specialist follow-up. This necessitates a service system capable of precise assessment and dynamic adjustment. Key indicators in our framework—including “conduct of baseline testing and reassessment,” “development of personalized intervention plans,” and “patient follow-up rate”—serve as critical quality checkpoints that go beyond merely documenting whether an intervention occurred, instead emphasizing whether it was standardized, tailored, and sustainable. Critically, MASLD care globally remains fragmented: patients are often identified only reactively upon elevated liver enzymes, without systematic metabolic risk screening or proactive behavioral support (25, 26). Lazarus et al. (27) have called for the formal inclusion of MASLD in national non-communicable disease strategies to enhance awareness among clinicians and the public, standardize care pathways, and strengthen cross-sectoral collaboration. In this context, our evaluation framework offers a quantifiable, replicable, and scalable model of integrated care. By institutionalizing the synergy between sports science and healthcare under the paradigm of physical and medical integration, it facilitates a strategic shift from reactive disease treatment to proactive health management. This not only enhances service quality at the facility level but also provides a robust evidence base and practical evaluation tool for future policy development—supporting the establishment of standardized MASLD prevention and management programs at regional and national scales.

This study has several limitations. First, to enhance clinical utility, the indicator selection prioritized representativeness, measurability, and implementability—potentially at the expense of theoretical comprehensiveness. Second, in the absence of large-scale real-world service data, the scoring criteria for individual indicators were primarily derived from expert judgment. Therefore, this study represents a preliminary developmental phase. Future research will conduct implementation studies in pilot hospitals.

## Conclusions

This study successfully developed an evaluation framework for lifestyle management of patients with MASLD under the concept of physical and medical integration. Structured across three dimensions—process, effect, and outcome—the framework demonstrates sound construct validity, scientifically derived weights, and strong operational feasibility. It directly addresses the central role of lifestyle intervention in MASLD management and aligns with the global shift toward integrated, multidisciplinary chronic care. This tool offers a practical and evidence-based resource for healthcare institutions to standardize MASLD lifestyle services, monitor service quality, optimize resource allocation, and advance the implementation of physical and medical integration. Future efforts should prioritize real-world application, psychometric validation (including reliability and validity testing), and iterative refinement to ensure the framework's adaptability and effectiveness across diverse settings—thereby bridging the critical gap between theoretical development and clinical implementation.

## Data Availability

The raw data supporting the conclusions of this article will be made available by the authors, without undue reservation.
